# A Thesis on Hospital Gangrene

**Published:** 1865-03

**Authors:** John E. Link


ARTICLE X.

### A THESIS ON HOSPITAL GANGRENE.

BY JOHN E. LINK.

Presented to the Faculty of the Chicago Medical College, February, 1865.

Hospital gangrene is characterized by a dark gray, pulpy slough, with inflammation of the surrounding tissues, presenting a circular form, with raised indurated edges, spreading rapidly. There is a discharge of dark, or yellow-greenish pus, with an offensive, pungent odor, and not to be mistaken, if once recognized. The patient complains of loss of appetite, an indisposition to sleep, has sharp, biting, or stinging pain, the pulse is quick and feeble, cheek flushed, tongue dry and furred. Sometimes, in the early stages of the disease, the patient complains of chilly sensations, followed by hot flashes, alternately. This is not so marked in the early as in the latter stages, where it has been permitted to run on for several days, and the patient is becoming exhausted. The disease is wholly local, the constitutional trouble being merely the result of the local, and at once disappears when it is checked. It generally attacks recent wounds, though not confined wholly to them. I have seen it, in a few instances, occur in old, indolent ulcers, and in suppurating wounds, that were closed to the size of a ten cent piece, or smaller. Stumps that are almost healed, are sometimes attacked, and the cicatrix destroyed, laying the flaps entirely open in a few days, if not checked. It confines itself, principally, to the integument and cellular planes, often dissecting the muscles and larger bloodvessels as nicely as if done with the scalpel. It sometimes, however, attacks these and converts them into a pulpy mass which sloughs away, and, in the latter case, causing dangerous hemorrhage. The bone is sometimes denuded and necrosed. In one case that came under my observation, the tendo achilles was divided, but all these, in the majority of cases is more the result of the remedies used than of the disease. The destruction of bone may generally be avoided by proper care. The cellular tissue is more readily affected and destroyed than the integument, thus causing saculation and burrowing of pus, and for this reason the wound should be thoroughly examined, by the probe or finger, to learn the extent of the disease. It is more liable to attack wounds from the fifth to the tenth day after the receipt of injury than at any subsequent period, or, after healthy granulations have once sprung up.

The causes of hospital gangrene are various. Among the most frequent are crowded or ill-ventilated wards, the effluvia arising from sinks, decaying vegetable or animal matter, or filth of any description. The crowding of too many suppurating wounds into one ward is a very frequent cause, as well as a lack of frequent and proper dressing, or using the same sponge and dresser for different patients. Where one patient is suffering f rom e disease, the others immediately around him, and even in different parts of the ward, are liable to be attacked, even where the most diligent sanitary rules are complied with, and all care taken to prevent actual contact by using separate dressings and dressers—for this reason, the cases suffering with the disease should be kept isolated from the other patients.

In the treatment of gangrene, the first and most necessary step, is the thorough cleansing of the wound, and as it is a very painful one, it is best to put the patient under the influence of chloroform or ether. The surgeon should arm himself with a pair of forceps, probe, scissors, scalpel, basin, and sponges. With the forceps and scissors, or scalpel (I generally prefer the latter), the surgeon proceeds to trim away the slough, and it is best in all cases to remove the skin as far as the disease has extended in the tissues beneath, to prevent the saculation of pus, giving free drainage, and on the first application, where the disease is first making its appearance, and the tissues are not entirely disorganized, it is best in these cases to make free use of the scalpel, and remove nearly, if not quite, the whole of the surrounding indurated tissue. After this is done, and the wound is thoroughly dried, apply some caustic, either nit. acid, bromine, or solution of chlorite of zinc, to destroy any remaining virus. Where this is done in the early stages of the disease, a second application is scarcely ever required, but where the disease has existed for a longer period, so that there is a large open sore, the whole face of which presents a dark gray, pulpy appearance, the management of the disease is more difficult, and may require several applications before it will be entirely checked. In all cases the wound should be thoroughly cleansed, by scraping with the handle of the scalpel or spatula, until the whole of the gangrenous slough is removed, and the healthy tissues beneath are reached. As a local application in these cases, I generally make use of the nitric acid. It is much more simple and easily applied, and also more penetrating than the bromine, finding its way to the most intricate interstices, while the bromine will not penetrate its own coagulum, and, owing to its volatility, is much more difficult to handle, and often, in the hands of those unaccustomed to using it, gives rise to very unpleasant circumstances, filling the room with its vapor, which, by no means, has a pleasant effect on the eyes and air-passages of those who come in contact with it; and even in the hands of the most practised, this cannot be wholly avoided.

The mode of applying nitric acid is simple. Tie a small piece of sponge, or a piece of soft cloth, to a small stick, making a swab or mop, which should be thoroughly saturated with the medicine, and applied to all parts of the wound which pours out a dark fluid, which should be removed with the sponge, and the application continued until the surrounding tissues lose their red, inflamed appearance, and become pale and soft, which will always be the case, if continued long enough, and if carried to this extent, it will very seldom be found necessary to repeat the treatment. The wound should now be dressed with simple water dressing—some prefer the charcoal and yeast poultice, which seems often to have a very good effect in removing the slough, and where they are frequently changed, and the wound cleansed, is very good treatment, but the wet cloths, which keep down the inflammation, and also keep the wound moist until healthy granulations spring up and remove the slough, which will occur in from three to four days, seems to be all-sufficient. After the slough has been removed, and there are any further signs of gangrene remaining, the application should be repeated, always giving ample time for the slough to be removed, unless the fetor can be detected, which is a certain indication that the disease is still lurking in some portion of the wound, and should at once be sought, giving it no time to spread, which it will do very rapidly if not checked, and thus all former treatment will be lost.

When the bromine is used, it is necessary to use some precaution to prevent the escape of the vapor. The bottle containing the medicine should be kept tightly closed until the wound is prepared, then it should be cautiously opened, and a small portion poured into a cup containing water. The bromine at once falls to the bottom, the water preventing the escape of vapor. A small glass or rubber syringe can now be inserted and a portion introduced, which can be applied to all parts of the wound, care being taken to introduce it well down into all the cavities. In this, much more care is required than where nitric acid is used. It is sufficient to pour the acid into the wound, when it will gravitate to almost all parts of the wound. Not so with the bromine, it must be introduced to all parts; the swab will answer for this purpose, but not so well as the syringe. After this has been done, the wound should be covered with a piece of cloth spread with simple cerate, to prevent the escape of the fumes arising from the bromine. This, when properly used, is an efficient remedy in the treatment of hospital gangrene, but under my observation is not as successful as nitric acid, owing to the difficulty of applying it, but where it can be thoroughly applied it will in all cases destroy the disease. The result of the treatment of gangrene was not as successful previous to the introduction of bromine by Dr. Goldsmith, in 1862, as it has been since, which is owing more, in all probability, to the free use of the scalpel in cleansing and thoroughly preparing the wound, than to any superior quality which it possessed over the acid, and solution of the chlorite of zinc, which had been in use. Where the bone is exposed, it should be kept coated over with simple cerate, to protect it from contact with the caustic. By proper care, in this way, necrosis, or caries, can generally be avoided. The constitutional treatment should be merely supportive, making free use of stimulants and tonics, with proper diet.

